# A Synthetic Human Kinase Can Control Cell Cycle Progression in Budding Yeast

**DOI:** 10.1534/g3.111.000430

**Published:** 2011-09-01

**Authors:** Megan J. Davey, Heather J. Andrighetti, Xiaoli Ma, Christopher J. Brandl

**Affiliations:** Department of Biochemistry, Schulich School of Medicine & Dentistry, University of Western Ontario, London, Ontario, Canada N6A 5C1

**Keywords:** DNA replication, kinase, S-phase, checkpoint, cell cycle

## Abstract

The DDK kinase complex, composed of Cdc7 and Dbf4, is required for S-phase progression. The two component proteins show different degrees of sequence conservation between human and yeast. Here, we determine that *Saccharomyces cerevisiae* bearing human *CDC7* and *DBF4* grows comparably to cells with yeast DDK under standard growth conditions. HsDrf1 (a second human Dbf4-like protein) does not support growth, suggesting that HsDbf4 is the true ortholog of ScDbf4. Both human subunits are required to complement yeast *cdc7Δ* or *dbf4Δ* due to the inability of human Cdc7 or Dbf4 to interact with the corresponding yeast protein. Flow cytometry indicates normal cell cycle progression for yeast containing human DDK. However, yeast containing human DDK is sensitive to long-term exposure to hydroxyurea and fails to sporulate, suggesting that human DDK substitutes for some, but not all, of yeast DDK’s functions. We mapped the region of Cdc7 required for species-specific function of DDK to the C-terminus of Cdc7 by substituting the yeast C-terminal 55 amino acid residues in place of the equivalent human residues. The resulting hybrid protein supported growth of a *cdc7Δ* strain only in the presence of *ScDBF4*. The strain supported by the hybrid *CDC7* was not sensitive to HU and formed tetrads. Together, our data indicate that DDK’s targeting of its essential substrate is conserved between species, whereas the interactions within DDK are species specific.

Dbf4-dependent kinase (DDK) is a serine/threonine kinase complex required for the initiation of DNA replication. Both the catalytic subunit (Cdc7) and the regulatory subunit (Dbf4) are essential for growth in budding and fission yeast (Brown and Kelly 1998; Brown and Kelly 1999; Hartwell *et al.* 1973; Kitada *et al.* 1992; Masai *et al.* 1995), acting throughout S-phase to fire origins (Bousset and Diffley 1998; Donaldson *et al.* 1998). In mammalian cells, depleting Cdc7 or Dbf4 adversely affects DNA replication and cell proliferation (Jiang *et al.* 1999; Kumagai *et al.* 1999). DDK requires Cdc7’s kinase activity for function. Its essential substrate is Mcm2-7, the catalytic core of the replicative helicase (Bruck and Kaplan 2009; Ohtoshi *et al.* 1997; Sheu and Stillman 2006; Sheu and Stillman 2010; Tsuji *et al.* 2006). Because of its importance in cell cycle progression, Cdc7 is being exploited as a therapeutic target in cancer (Montagnoli *et al.* 2010; Sawa and Masai 2009; Swords *et al.* 2010). In addition to its essential role in DNA replication initiation, DDK functions in the S-phase checkpoint, (Costanzo *et al.* 2003; Dolan *et al.* 2010; Duncker and Brown 2003; Fung *et al.* 2002; Gabrielse *et al.* 2006; Matsumoto *et al.* 2010; Njagi and Kilbey 1982; Ogi *et al.* 2008; Pessoa-Brandao and Sclafani 2004; Tsuji *et al.* 2008; Weinreich and Stillman 1999), mitotic exit (Miller *et al.* 2009), and meiosis (Katis *et al.* 2010; Lo *et al.* 2008; Marston 2009; Nakamura *et al.* 2002; Valentin *et al.* 2006; Wan *et al.* 2008).

Yeast and human Cdc7 are well conserved within the kinase family subdomains but much less so in the insertions between the subdomains [Figure 1A; (Hanks *et al.* 1988; Masai *et al.* 1995)]. Dbf4 (also called ASK for activator of S-phase kinase in human cells) contains only three short conserved regions, termed N, M, and C (Masai and Arai 2000; Ogino *et al.* 2001). A second Dbf4-like subunit found in many metazoans, Drf1 (Dbf4-related factor 1, also called DBF4B or ASKL1) forms an independent kinase complex with Cdc7 (Montagnoli *et al.* 2002; Takahashi and Walter 2005; Yoshizawa-Sugata *et al.* 2005). Drf1 (DBF4B) should not be confused with DIAP1, also referred to as DRF1 (diaphanous-related formin 1) in humans. Depletion of Drf1 perturbs the cell cycle in human cells, but the phenotype is less severe than depletion of Cdc7 or Dbf4 (Yoshizawa-Sugata *et al.* 2005). In *Xenopus*, Drf1 and Dbf4 are developmentally regulated, with each being essential at different life stages (Takahashi and Walter 2005). A second Dbf4 subunit is not found in *S. cerevisiae*.

**Figure 1:**
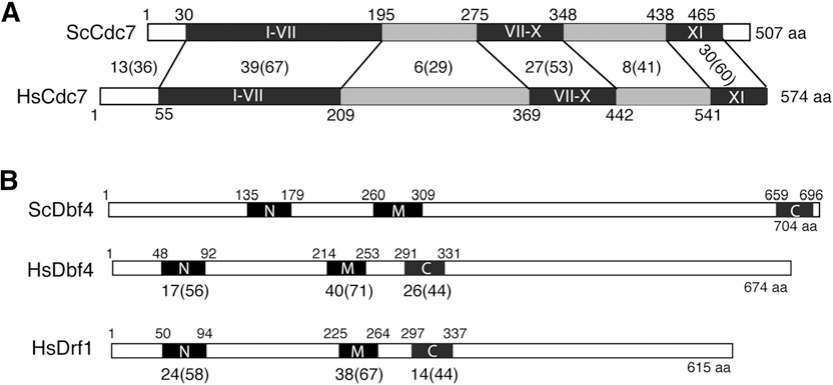
Human and yeast Cdc7 and Dbf4/Drf1. A) Comparison of the primary structure of Cdc7 from human (HsCdc7) and yeast (ScCdc7). The positions of conserved subdomains found in the eukaryotic protein kinase superfamily are indicated by black boxes. Kinase inserts, characteristic of the Cdc7 family, are indicated by gray boxes. The percent identity (similarity) in each region is indicated. B) Comparison of human Dbf4, yeast Dbf4, and human Drf1 primary structures. Black boxes mark the positions of the conserved Dbf4 motifs. The percent identity (similarity) of HsDbf4 and HsDrf1 with ScDbf4 in the motif regions is indicated under the schematics.

To evaluate the degree of functional conservation between human and budding yeast DDK with the goal of providing a genetically amenable system to study the structure and function of human DDK, we tested whether human Cdc7, Dbf4, and/or Drf1 substitute for DDK’s essential activity in *S. cerevisiae*. We find that human (Hs) *CDC7* and *DBF4*, but not *DRF1*, complement deletion of the yeast genes. Both *HsCDC7* and *HsDBF4* must be present for complementation, a result that agrees with a lack of interaction between the yeast and human proteins. Yeast strains with human DDK are sensitive to hydroxyurea (HU), a phenotype associated with some Dbf4 mutations (Gabrielse *et al.* 2006; Harkins *et al.* 2009; Jones *et al.* 2010), and do not form tetrads. By substituting the C-terminal 55 amino acid residues of yeast Cdc7 in place of the C-terminal 52 amino acid residues of human Cdc7, we generated a hybrid Cdc7 molecule that functions with ScDbf4 but not HsDbf4. Interestingly, changing the Dbf4 specificity of HsCdc7 to ScDbf4 relieves the HU sensitivity, suggesting that resistance to HU is provided by Dbf4. We thus demonstrate that the recognition of essential targets is conserved between DDK of different species despite the lack of cross-species interaction between the subunits. The results have implications for the study of Cdc7 and Dbf4 as targets for drug therapies and in the development of synthetic genomes.

## Materials and Methods

### Plasmids

All molecules were amplified by PCR using *Pwo* polymerase (Roche) and the primers in Table S1. A 3 kb *Sal*I-*Sbf*I fragment containing the promoter and coding regions of *ScDBF4* was cloned into the *URA3* centromeric plasmid YCplac33 (Gietz and Sugino 1988). *ScCDC7*, *HsCDC7*, and *HsDBF4* were expressed from the *ScCDC7* promoter, inserted as *Sma*I-*Sal*I fragments into YCplac33 (*URA3-CEN*) or YCplac111 [*LEU2-CEN*; (Gietz and Sugino 1988)]. Coding sequences were inserted using an *Nde*I site placed at the ATG start codon and downstream *Hin*dIII (*ScCDC7*), *Sbf*I (*HsCDC7*, *HsDBF4*), or *Nde*I sites (*HsDRF1*). cDNA clones for human *CDC7*, *DBF4*, and *DRF1* were purchased from Open Biosystems (accession numbers BC11044, BC047693, and MHS1011-74961). Human genes were transferred to the 2 µ *LEU2* episomal plasmid YEplac181 (Gietz and Sugino 1988) using *Sac*I-*Sbf*I (*HsCDC7*, *HsDBF4*) or *Sma*I-*Bam*HI (*DRF1*). The *LEU2* markers of YCplac111-*HsCDC7* and YEplac181-*HsCDC7* were switched to *HIS3* with pLH7 (Cross 1997), yielding YCplac111h-*HsCDC7* and YEplac181h-*HsCDC7*. The *URA3* marker on YCplac33-*ScDBF4* was switched to *LEU2* with pUL9 (Cross 1997). *Myc*^9^-tagged versions of the human proteins were inserted into YCp88-*myc^9^* (Hoke *et al.* 2008) as *Not*I-*Sac*I or *Not*I-*Eco*RI fragments. A plasmid encoding both *ScCDC7* and *ScDBF4* (YCplac33-*ScCDC7-ScDBF4*) was constructed by inserting a *Sma*I-*Xba*I fragment containing the *ScCDC7* promoter and ORF (amplified using MD405 and MD438) into YCplac33*-ScDBF4*. C-terminal fragments of ScCdc7 were substituted in place of the corresponding HsCdc7 regions using gene splicing by overlapping extension PCR (Horton *et al.* 1990) and inserted as *Nde*I-*Sbf*I fragments into YEplac181 with the *ScCDC7* promoter to yield YEplac181-*CDC7-S1*, YEplac181-*CDC7-S2*, and YEplac181-*CDC7-S3*. The *LEU2* marker on these plasmids was switched to *HIS3* using pLH7 (Cross 1997).

### Yeast strains

Strains are listed in Table S2. Heterozygous deletion strains (BY23713, *cdc7∆0*; BY23988, *dbf4∆0*) (Giaever *et al.* 2002) and TAP-tagged Cdc7 and Dbf4 strains (Ghaemmaghami *et al.* 2003) were purchased from Open Biosystems. To generate haploid deletion strains complemented by plasmid copies, BY23713 was transformed with YCplac33-*ScCDC7*, and BY23988 with YCplac33-*ScDBF4*. Transformants were sporulated and G418 resistant colonies selected (MDY95: *MAT*a, *cdc7∆0*, YCplac33-*ScCDC7*; CY4104: *MAT*α, *dbf4∆0*, YCplac33-*ScDBF4*). To generate a diploid strain deleted for *cdc7* and *dbf4*, *KanMX* in CY4104 was switched to *NatMX* by transformation with linearized p4339 (Tong and Boone 2006), creating CY4178. CY4178 was mated to MDY95 and the *URA3* plasmids shuffled out, creating CY4348 (*cdc7::KanMX dbf4::NatMX*). CY4348 was then transformed with YEplac181*-HsDBF4* and YCplac111h-*HsCDC7* or YEplac181h-*HsCDC7*, sporulated and His+, Leu+, Ura−, G418, and ClonNAT resistant colonies identified to generate CY4240 and CY4242. To generate a double deletion strain complemented by *ScCDC7* and *ScDBF4*, YCplac111-*ScCDC7* was shuffled into MDY95 to yield MDY195. Diploids from a MDY195 and CY4178 mating were sporulated. Leu+, Ura+, and *MAT*a spore colonies that were G418 and ClonNAT resistant were identified (CY4481: *cdc7∆0 dbf4∆0*; YCplac111-*ScCDC7*, YCplac33-*ScDBF4*). A strain deleted for both *cdc7* and *dbf4* containing YCplac33-*ScCDC7-ScDBF4* was created by transforming CY4240 (*MAT*α *cdc7::KanMX dbf4::NatMX* YCplac111h-*HsCDC7* YCplac181-*HsDBF4*) with YCplac33-*ScCDC7-ScDBF4*. After several passages in YPD, colonies that were Ura+, Leu−, His−, and 5-FOA sensitive were screened to yield MDY214 (*MAT*α *cdc7::KanMX dbf4::NatMX* YCplac33-*ScCDC7-ScDBF4*). For homozygous diploid strains deleted at *cdc7* and *dbf4*, CY4242 (*MAT*a *cdc7::KanMX dbf4::NatMX* YCplac181h-*HsCDC7* YCplac181-*HsDBF4*) and MDY214 were mated to create MDY270. Treatment with 5-FOA generated a strain containing only human DDK (CY5628; *MAT*a/*MAT*α *cdc7::KanMX dbf4::NatMX* YCplac181h-*HsCDC7* YCplac181-*HsDBF4*). After several passages of MDY270 through YPD, Ura+, Leu−, and His− colonies were identified to generate CY5627 (*MAT*a/*MAT*α *cdc7::KanMX dbf4::NatMX* YCplac33-*ScCDC7-ScDBF4*). CY5627 was transformed with YEplac181h-*CDC7-S1*, and YCplac33L-*ScDBF4*, His+, and Leu+ colonies selected, and then the strain (MDY317) was plated on 5-FOA to yield MDY318.

### Cell cycle arrest and flow cytometry

Yeast strains CY4240 (HsDDK) and CY4481 (ScDDK) were grown in YPD to an OD_600_ of ∼0.6, incubated in 100 mM hydroxyurea (Sigma-Aldrich) for 2 h at 30°, washed twice, and then resuspended in fresh YPD. Aliquots were removed at the indicated times, pelleted, and resuspended in 70% ethanol. Cell sorting was performed on a FACSCalibur (BD Biosciences) by the London Regional Flow Cytometry Facility.

### Cdc7 and Dbf4 interactions

Log phase cells (4 × 10^10^ cells) were harvested by centrifugation, washed with ice-cold water, and resuspended in 3 ml lysis buffer [25 mM Tris-HCl pH 8.0, 150 mM NaCl, 0.1 mM EDTA pH 8.0, and 0.1% (v/v) NP40]. Cell extracts were made as described by Schultz *et al.* (1991). After centrifugation, the supernatant (∼4 ml, 15–20 mg protein/ml) was mixed with 50 µl Epoxy M270 Dynabeads (Invitrogen) coupled to rabbit IgG (∼7 mg IgG/ml beads). After 2 h at 4°, beads were collected and the supernatant removed. The beads were washed twice with 1 ml of lysis buffer, once in 200 µl lysis buffer, and then incubated in 50 µl SDS loading dye at 90° for 5 min. The elutions were analyzed by Western blotting. Primary antibodies were anti-ScCdc7 yN-18 and anti-ScDbf4 yA-16 (Santa Cruz Biotechnology) and monoclonal anti *c-myc* (Sigma-Aldrich). Rabbit anti-goat and goat anti-mouse antibodies coupled to horseradish peroxidase were from Sigma-Aldrich. Detection using chemiluminscence followed the manufacturer’s instructions (SuperSignal West Pico Kit; Pierce Biotechnology).

## Results

### Functional complementarity of human and yeast DDK

**Figure 2:**
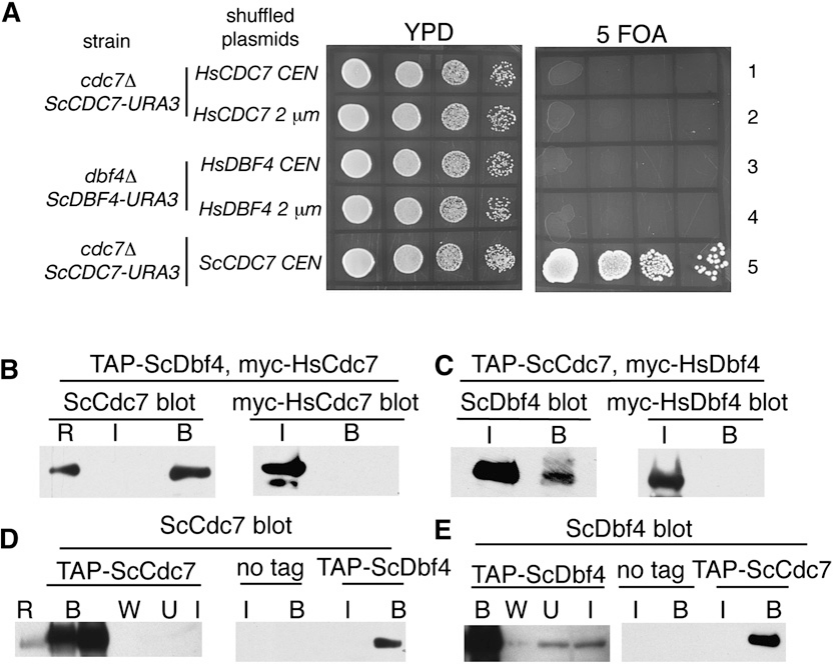
Human *CDC7* and *DBF4* do not interact with their yeast counterparts. A) Haploid *cdc7* (rows 1, 2, and 5) or *dbf4* (rows 3 and 4) deletion strains [BY23713, *cdc7∆0*; BY23988, *dbf4∆0*; (Giaever *et al.* 2002)] transformed with the cognate yeast gene on a *URA3* encoding plasmid (Gietz and Sugino 1988) were transformed with the indicated *CEN* or 2 µ plasmids (Gietz and Sugino 1988) encoding copies of *HsCDC7*, *HsDBF4*, or *ScCDC7*. Cell suspensions were diluted and then spotted on YPD (left) or 5-FOA–containing media (right) and grown at 30° for two and three days, respectively. B) TAP-ScDbf4–associated proteins from extracts containing the indicated proteins were isolated using rabbit IgG coupled Epoxy M270 Dynabeads and then probed for the presence of ScCdc7 or *myc*^9^-HsCdc7 by Western blotting. “R” indicates recombinant yeast Cdc7 [∼100 ng; (Stead *et al.* 2011)], “I” the input extract (∼1/100 of total), and “B” bound protein. C) A similar experiment to probe for the association of ScDbf4 and *myc*^9^-HsDbf4 with TAP-ScCdc7. D and E) Control experiments showing that the TAP-tagged proteins are enriched by the IgG beads (left panels) and that binding of ScCdc7 (D) or ScDbf4 (E) to the beads depends on the presence of TAP-ScDbf4 or TAP-ScCdc7, respectively. The “no tag” strain is BY4741. “W” is the wash.

To address the degree of functional conservation between yeast and human DDK subunits, we tested whether the individual human subunits were able to substitute for their yeast counterparts. We expressed *HsCDC7* or *HsDBF4* from centromeric and 2 µ plasmids (Gietz and Sugino 1988) and examined their ability to support viability by plasmid shuffling. The initial analysis was performed with *CDC7* in which a haploid yeast strain deleted for *cdc7* (Giaever *et al.* 2002) and complemented by a *URA3* plasmid encoding *ScCDC7* was transformed with either low copy or multicopy plasmids encoding *HsCDC7*. Cells were plated on 5-FOA where viability requires that the human gene substitutes for its yeast ortholog. As shown in Figure 2A (rows 1, 2), neither the low copy nor the multicopy plasmid encoding *HsCDC7* supported viability of the *cdc7Δ* strain. In similar tests, *HsDBF4* did not support growth of *dbf4Δ* (Figure 2A, rows 3, 4).

The lack of complementation could be explained by the inability of HsCdc7 and HsDbf4 to interact with their yeast counterparts. To test this possibility, we compared the interaction of ScCdc7 and *myc*^9^-tagged HsCdc7 with ScDbf4 fused to a tandem affinity purification (TAP) tag (Rigaut *et al.* 1999). Proteins that interacted with affinity-purified TAP-ScDbf4 were detected by Western blotting. As expected, we detected a robust signal for ScCdc7 in the fraction eluting with TAP-ScDbf4 (Figure 2B, left, D). In contrast to ScCdc7, *myc^9^*-HsCdc7 was not associated with TAP-ScDbf4, even though there was a strong signal for this protein in the cell lysate (Figure 2B, right). Similar experiments with TAP-ScCdc7 and *myc^9^*-HsDbf4 did not detect an interaction between ScCdc7 and HsDbf4 (Figure 2C, E). A reciprocal experiment in which antibodies to the human proteins were used also did not identify a complex of yeast and human proteins (supporting information, Figure S1). We therefore concluded that HsCdc7 and HsDbf4 fail to interact with ScDbf4 and ScCdc7, respectively.

**Figure 3:**
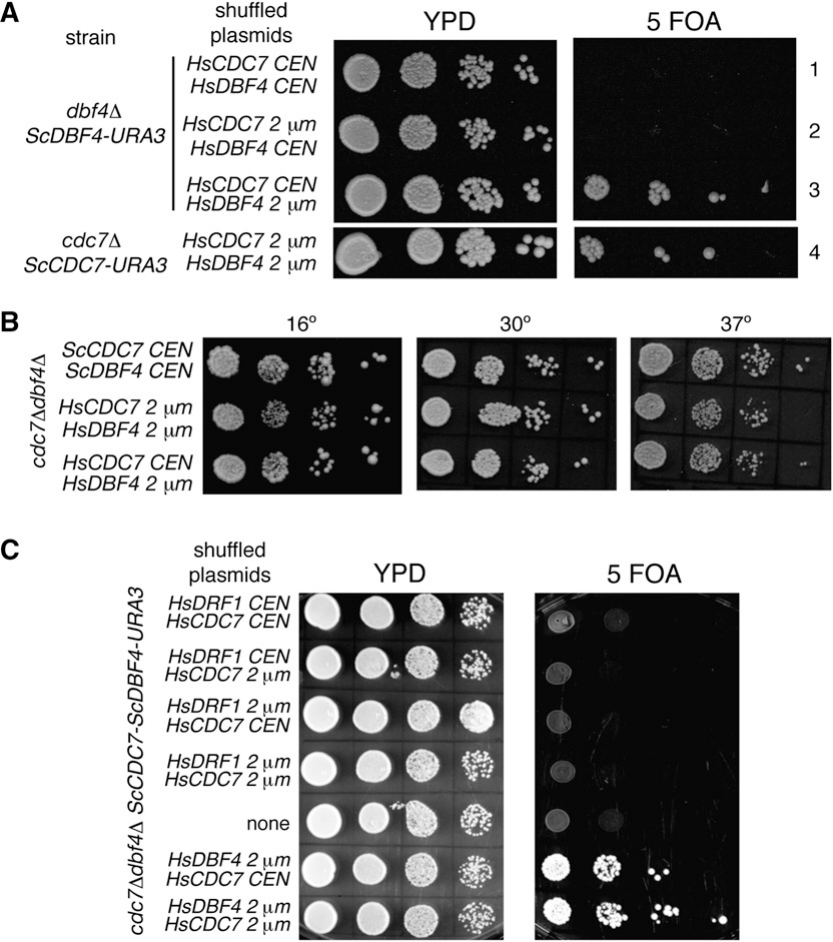
Complementation of *cdc7Δdbf4Δ* by human DDK. A) Haploid strains deleted for *dbf4* or *cdc7* (Giaever *et al.* 2002) and bearing the indicated plasmids encoding yeast and human *CDC7* and/or *DBF4* were spotted on YPD and 5-FOA plates and then grown at 30° for two and three days, respectively. B) Haploid strains deleted for *cdc7* and *dbf4* (*cdc7::KanMX dbf4::NatMX*) and bearing the indicated plasmids encoding yeast or human *CDC7* and *DBF4* were spotted on YPD plates and then grown at 16° for seven days, or 30° or 37° for two days. C) MDY214 was transformed with *CEN* or multicopy plasmids encoding *HsDRF1* and *HsCDC7* as indicated. Five thousand cells and 10-fold serial dilutions were plated on YPD and 5-FOA and grown at 30° for one and three days. As a control, MDY214 transformed with *HsCDC7* and *HsDBF4* is also shown (bottom rows).

Since a cross-species DDK complex did not form, we wondered whether supplying both human genes would allow complementation of *cdc7Δ* or *dbf4Δ*. To test this, we repeated the plasmid shuffling, supplying copies of both *HIS3-HsCDC7* and *LEU2-HsDBF4* (Figure 3A). Strains containing *HsCDC7* and multicopy *HsDBF4* were viable regardless of whether the yeast strain was deleted in *cdc7* or *dbf4* (Figure 3A, rows 3, 4). *HsCDC7* was functional when expressed from either centromeric or multicopy plasmids. In contrast, only the multicopy plasmid bearing *HsDBF4* complemented.

To determine whether the presence of *ScDBF4* or *ScCDC7* contributed to the activity of human DDK, we constructed a diploid strain (CY4348) heterozygous for deletion of both *CDC7* (*cdc7::KanMX*) and *DBF4* (*dbf4::NatMX*) in which the deletions provided resistance to G418 and ClonNAT, respectively. CY4348 was transformed with plasmids encoding *HsCDC7* and *HsDBF4*. After sporulation and tetrad dissection, spore colonies that were both G418 and ClonNAT resistant were obtained. These spore colonies were all His+ (*HsCDC7*) and Leu+ (*HsDBF4*), indicating that HsDDK substitutes for ScDDK’s essential function. PCR analysis confirmed that neither *ScCDC7* nor *ScDBF4* were present in the HsDDK strains (Figure S2). No major differences in growth between cells containing plasmid-encoded human or yeast DDK were noted at 16°, 30°, or 37° (Figure 3B). In addition, cells bearing HsDDK grew at the same rate in liquid media as strains containing plasmid-encoded ScDDK (Figure S3). Thus, we concluded that a complex of HsCdc7 and HsDbf4, comprising HsDDK, substitutes for ScDDK and that this complex is sufficient to support viability in a temperature-independent manner.

### Human Drf1 in yeast cells

Many metazoans contain a second homolog to Dbf4 called Drf1 (Furukohri *et al.* 2003; Montagnoli *et al.* 2002; Yoshizawa-Sugata *et al.* 2005). Depletion of Drf1 from cultured human cells interferes with cell cycle progression, but the phenotype is not as severe as depletion of Cdc7 or Dbf4 (Yoshizawa-Sugata *et al.* 2005). To determine whether *HsDRF1* would support viability of yeast in place of *HsDBF4*, we cotransformed *HsDRF1* on either *LEU2* 2 µ or *CEN* plasmids with *HsCDC7* into the *cdc7Δdbf4Δ* strain maintained by a *URA3* plasmid encoding *ScCDC7* and *ScDBF4*. Spotting the transformants on 5-FOA determined that *HsDRF1* (with *HsCDC7*) was unable to complement *cdc7Δdbf4Δ* (Figure 3C) under conditions in which *HsDBF4* supports viability. Western blotting of a strain containing myc-tagged Drf1 indicated that it was expressed (Figure S4). These results suggest that *HsDBF4* is the true ortholog of *ScDBF4*.

### Human DDK supports yeast cell cycle progression

**Figure 4:**
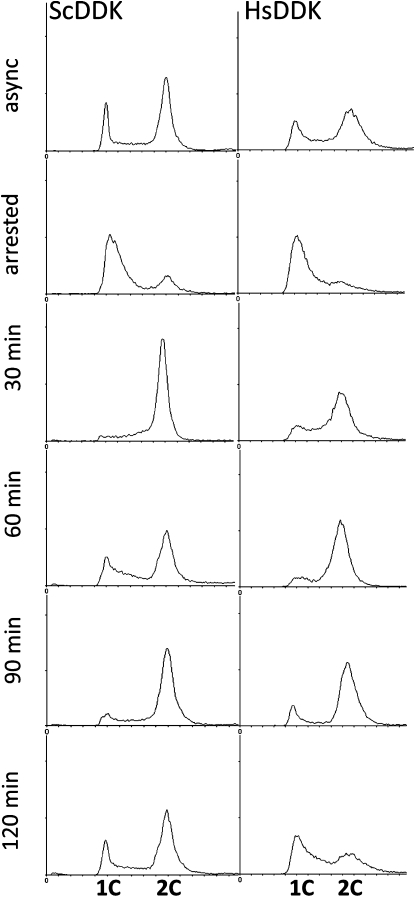
Effect of human DDK on the cell cycle progression of yeast. The progression of the cell cycle after arrest and release with HU was examined for a strain containing HsDDK (CY4240; *cdc7::KanMX dbf4::NatMX* YCplac111h-*HsCDC7* YCplac181-*HsDBF4*) or plasmid-encoded ScDDK (CY4481; *cdc7::KanMX dbf4::NatMX*, YCplac111-*ScCDC7*, YCplac33-*ScDBF4*). Log phase cells were incubated in 100 mM hydroxyurea (Sigma-Aldrich) for 2 h at 30°, washed twice, and then resuspended in fresh YPD. Aliquots were removed at the indicated times and subsequently examined for DNA content by FACS. The top panel is the analysis of asynchronously growing cells. The positions of cells containing 1C and 2C DNA content are indicated below.

To address whether the cell cycle proceeds normally in yeast supported by human DDK, we performed flow cytometry on asynchronous cultures and synchronized cells arrested in mid–S phase using HU, an inhibitor of ribonucleotide reductase that induces the S-phase checkpoint and prevents firing of late origins. In asynchronous cultures, there were similar ratios of replicated and unreplicated cells (2C and 1C, respectively) for the human DDK and yeast DDK strains (Figure 4, top panel). Flow cytometry was also performed on cells arrested with HU, and then at different times after release from the block. Strains bearing HsDDK showed a similar pattern, but they were slightly slower in progressing through the cell cycle than cells with ScDDK after release from HU, particularly in the initial recovery from HU (Figure 4, 30 min time point).

### Response of human DDK to genotoxic agents in yeast cells

**Figure 5:**
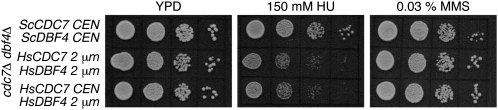
Effects of human DDK on the response of yeast to genotoxic agents. The haploid strain (*cdc7::KanMX dbf4::NatMX*) deleted for both *cdc7* and *dbf4* and bearing the indicated plasmids encoding yeast or human *CDC7* and *DBF4* were spotted (5000 cells and 10-fold serial dilutions) on YPD plates as well as YPD plates containing 150 mM HU or 0.03% MMS. Cells were grown for two days at 30°.

DDK is important for yeast to respond to replicative stress. Mutations of Dbf4 result in sensitivity to genotoxic agents (Fung *et al.* 2002; Gabrielse *et al.* 2006; Harkins *et al.* 2009; Njagi and Kilbey 1982; Varrin *et al.* 2005). To examine whether HsDDK substitutes for ScDDK in this regard, we grew yeast strains containing human or yeast DDK under conditions of replicative stress. As shown in Figure 5, strains containing HsDDK were more sensitive to HU compared with strains containing ScDDK. The sensitivity to HU was independent of *HsCDC7* copy number. Note that in these experiments, the exposure to HU was continuous, whereas in the flow cytometry experiments the exposure to HU was relatively brief. A similar phenotype was observed with *S. cerevisiae* Dbf4 mutants (Jones *et al.* 2010). No changes in growth were noted with HsDDK on plates containing methyl methanesulfonate (MMS), a DNA-damaging agent (Figure 5).

### Human DDK does not support sporulation of yeast

**Figure 6:**
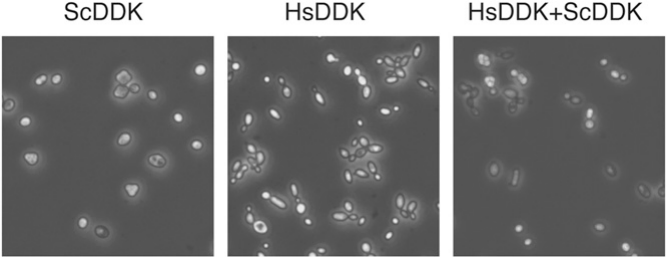
HsDDK diploids do not support meiosis. Light microscope images (400×) of diploid strains deleted at *cdc7* and *dbf4* with plasmid-encoded HsDDK and/or ScDDK. Images are of strains after at least five days incubation in 1% potassium acetate at 30°.

DDK is required for recombination and segregation events during meiosis in yeast (Marston 2009). To examine whether human DDK can substitute for yeast DDK during meiosis, we generated diploid homozygous *cdc7Δdbf4Δ* strains bearing either ScDDK (CY5627) or HsDDK (CY5628) on plasmids. Each of the diploid strains was incubated in 1% potassium acetate for 5 to 7 days, and then examined for tetrads. Ten out of 10 sporulation cultures of the ScDDK strain contained tetrads, whereas none of the HsDDK sporulation cultures contained tetrads. Furthermore, the HsDDK cells had an abnormal appearance (Figure 6). The lack of tetrads with HsDDK was consistent with previous studies using mutations in DDK (Lo *et al.* 2008; Matos *et al.* 2008). The inhibition of sporulation by HsDDK was recessive as a strain with both human and yeast DDK (MDY270) contained tetrads after incubation in potassium acetate (Figure 6).

### Region of Cdc7 required for species-specific formation of functional DDK

**Figure 7:**
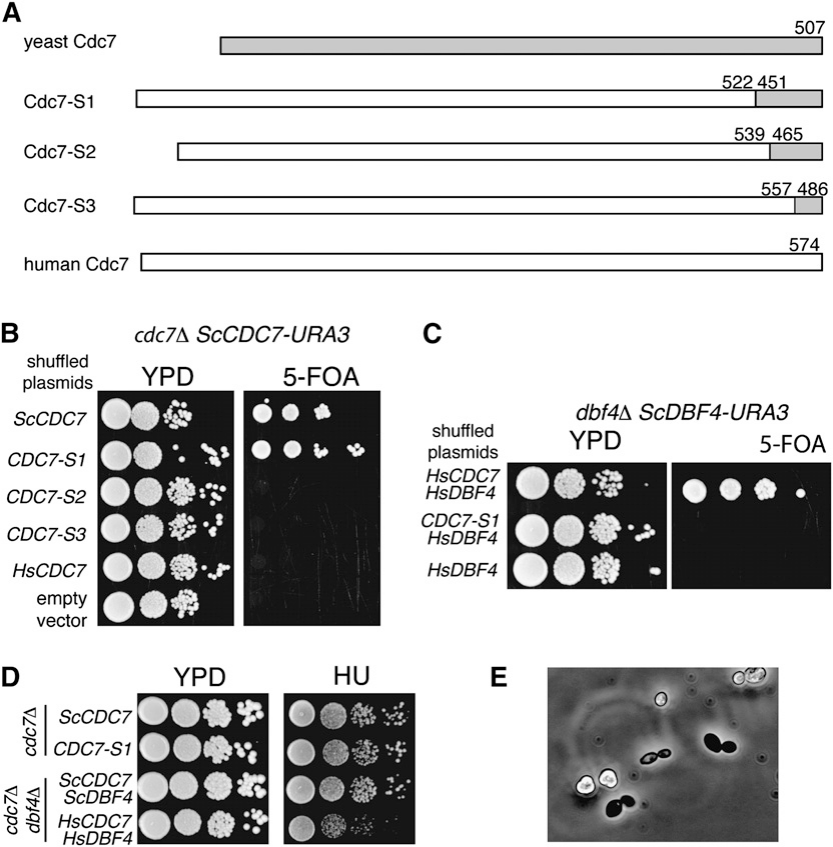
Definition of the species-specific interaction region in Cdc7. A) Positions of each swap are shown, as are schematics of the full-length proteins, with human sequences in white and yeast sequences in gray. B) Haploid *cdc7Δ* strains supported with *URA3-ScCDC7* were transformed with *LEU2* plasmids encoding *HsCDC7*, *ScCDC7*, or the indicated yeast-human *CDC7* chimeras. The strains were diluted serially and then spotted on 5-FOA. C) Haploid *dbf4* deletion strain with *URA3-ScDBF4* was transformed with both *LEU2-HsDBF4* and a *HIS3* 2 µ plasmid encoding wild-type *HsCDC7* or a chimera. The strains were serially diluted and spotted on YPD and 5-FOA. D) Strains deleted at *cdc7* complemented by *ScCDC7* or *CDC7-S1* were diluted and spotted on YPD or YPD containing 150 mM HU. For comparison, strains deleted at both *cdc7* and *dbf4* containing either ScDDK or HsDDK were also spotted. E) Image of a tetrad after incubation of MDY318 in potassium acetate (400×).

In *S. cerevisiae*, the region of Cdc7 that interacts with ScDbf4 maps to a 55 amino acid residue region at the C-terminus of ScCdc7 using yeast two-hybrid analysis (Jackson *et al.* 1993). While this region interacts with Dbf4, no information is available on whether the C-terminal 55 amino acid residues of ScCdc7 are sufficient to direct formation of a functional DDK complex. To address this, we created an allele in which amino acid residues 523-574 of HsCdc7 were replaced with residues 450-507 of ScCdc7 (Figure 7A). This chimeric allele was expressed on a *LEU2* 2 µ plasmid that was then shuffled into MDY99 (*cdc7::KanMX ScCDC7-URA3*). Cells containing the hybrid protein (Cdc7-S1) grew as well as cells containing ScCdc7 on 5-FOA (Figure 7B). In contrast, when shorter regions of the C-terminus (Cdc7-S2 and Cdc7-S3 in Figure 7A) were substituted, the hybrid proteins were unable to support viability of a *cdc7Δ* strain. Western blotting of the swapped constructs, tagged with myc^9^, indicated that the proteins were expressed (Figure S5). These results thus identify the C-terminal 55 amino acid residues as critical in defining the species specificity of *S. cerevisiae* Cdc7.

To determine whether replacement of the C-terminal 52 amino acid residues of HsCdc7 with yeast sequence interferes with the interaction between HsCdc7 and HsDbf4, we tested whether CDC7-S1 forms functional DDK with HsDbf4. As shown in Figure 7C, *CDC7-S1* did not support growth of a *dbf4Δ HsDBF4* strain, identifying a key role of the C-terminal 52 amino acid residues of HsCdc7 in species-specific formation of functional DDK, consistent with the requirement of amino acid residues 566-572 for interaction with HsDbf4 (Kitamura *et al.* 2011). We were unable to examine whether the shorter swaps (Cdc7-S2 and Cdc7-S3) support viability of *dbf4Δ HsDBF4* because we did not obtain transformants with these plasmids.

As shown above, strains containing HsDDK are sensitive to HU. To address whether this HU sensitivity is due to *HsCDC7* or *HsDBF4*, we compared the growth of the *cdc7Δ* strains containing plasmid-encoded *ScCDC7* or *CDC7-S1* and *ScDBF4* on YPD and HU. The *CDC7-S1* strain grew comparably to the *ScCDC7* strain on both media, suggesting that the Dbf4 subunit provides the target specificity for the response to HU.

We also examined whether *CDC7-S1* supports sporulation; recall that the diploid strain with HsDDK does not form tetrads. We transformed *CDC7-S1 HIS3* and *ScDBF4LEU2* into CY5627 (*cdc7Δ/cdc7Δdbf4Δ/dbf4Δ ScCDC7-ScDBF4-URA3*), treated the cells with 5-FOA to remove *ScCDC7*, and then incubated the resulting strain (MDY318) in potassium acetate. As seen in Figure 7E, tetrads were detected, suggesting that the defect in sporulation with HsDDK was in HsDbf4.

## Discussion

We established that the essential function of DDK is conserved between yeast and humans despite limited sequence conservation between the Dbf4 orthologs. These findings also demonstrate that it is their function as a complex rather than independent subunits that is essential for viability. Of note, in this two-component enzyme, the structure required for targeting of DDK to its essential substrates is conserved, but the regions required for interaction of the component proteins, Cdc7 and Dbf4, are not.

In yeast, the Mcm4 subunit of Mcm2-7, the replicative helicase, is the essential substrate of DDK. Phosphomimetic substitutions at the DDK target sites of Mcm4 bypass the requirement for *CDC7* and/or *DBF4* (Randell *et al.* 2010; Sheu and Stillman 2010). Targeting of Mcm4 is thought to occur through the Cdc7 subunit, which recognizes a “docking” site in a conserved region of Mcm4 (Sheu and Stillman 2006). Other Mcms are also substrates for DDK, including Mcm2 and Mcm6. Phosphorylation of Mcm6, with phosphorylation of Mcm4, is important for the initiation of DNA replication (Randell *et al.* 2010). Mutation of DDK-phosphorylation sites in Mcm2 does not affect viability; however, phosphorylation of Mcm2 by DDK may be important in the response to DNA damage (Randell *et al.* 2010; Stead *et al.* 2011).

### Checkpoint functions of DDK

The sensitivity of the HsDDK-containing strain to HU but not MMS indicates that HsDDK can perform some, but not all, of ScDDK’s functions in response to stress and is consistent with reports indicating that different regions of Dbf4 are required for the response to HU and MMS (Fung *et al.* 2002; Gabrielse *et al.* 2006). Interestingly, the strain maintained by Cdc7-S1 and ScDbf4 was resistant to HU, implicating Dbf4 as the important subunit within DDK for providing resistance to HU, although it is still formally possible that the C-terminal 55 residues of ScCdc7 are important for resistance to HU.

The ability of ScDbf4 to confer resistance to HU suggests that it may have important roles in defining target molecules during the response to HU. Indeed, recognition of Mcm2 by DDK is thought to occur through the Dbf4 subunit in yeast. Mutations in Dbf4 that interfere with Mcm2 interaction include those that lead to sensitivity to HU (Jones *et al.* 2010). Interestingly, the sensitivity of HsDDK strains to HU is suppressed by a version of Mcm2 with phosphomimetic glutamic acid residue substitutions at the DDK target sites S164 and S170 (Stead *et al.* 2011). The targeting of Mcm2 by Dbf4 contrasts with the observation that Mcm4 recognition occurs through Cdc7 (Sheu and Stillman 2006; Bernard Duncker, personal communication) and provides an explanation for conservation of the sequences required to target Mcm4 but not Mcm2. Interestingly, ScDbf4 (with Cdc7-S1) also rescued the defect in sporulation with HsDDK. Not surprisingly, interaction of ScDDK with Cdc5, required for meiosis, is mediated by ScDbf4 (Chen and Weinreich 2010; Matos *et al.* 2008; Miller *et al.* 2009).

### Species-specific Cdc7-Dbf4 interaction

Jackson *et al.* (1993) found the C-terminal 55 amino acid residues of ScCdc7 sufficient for interaction with Dbf4 in a yeast two-hybrid assay. We determined that the C-terminal 55 amino acid residues of yeast Dbf4 are necessary and sufficient for species-specific formation of functional DDK. Swapping shorter portions of the C-terminus did not support formation of a functional DDK with ScDbf4, suggesting that the region encompassing amino acid residues 449-561 of ScCdc7 is required for interaction with ScDbf4. The C-terminus of HsCdc7 is likely required for formation of functional DDK with HsDbf4, as replacement of the human C-terminal 52 amino acid residues with yeast sequence was not functional with HsDbf4.

**Figure 8:**
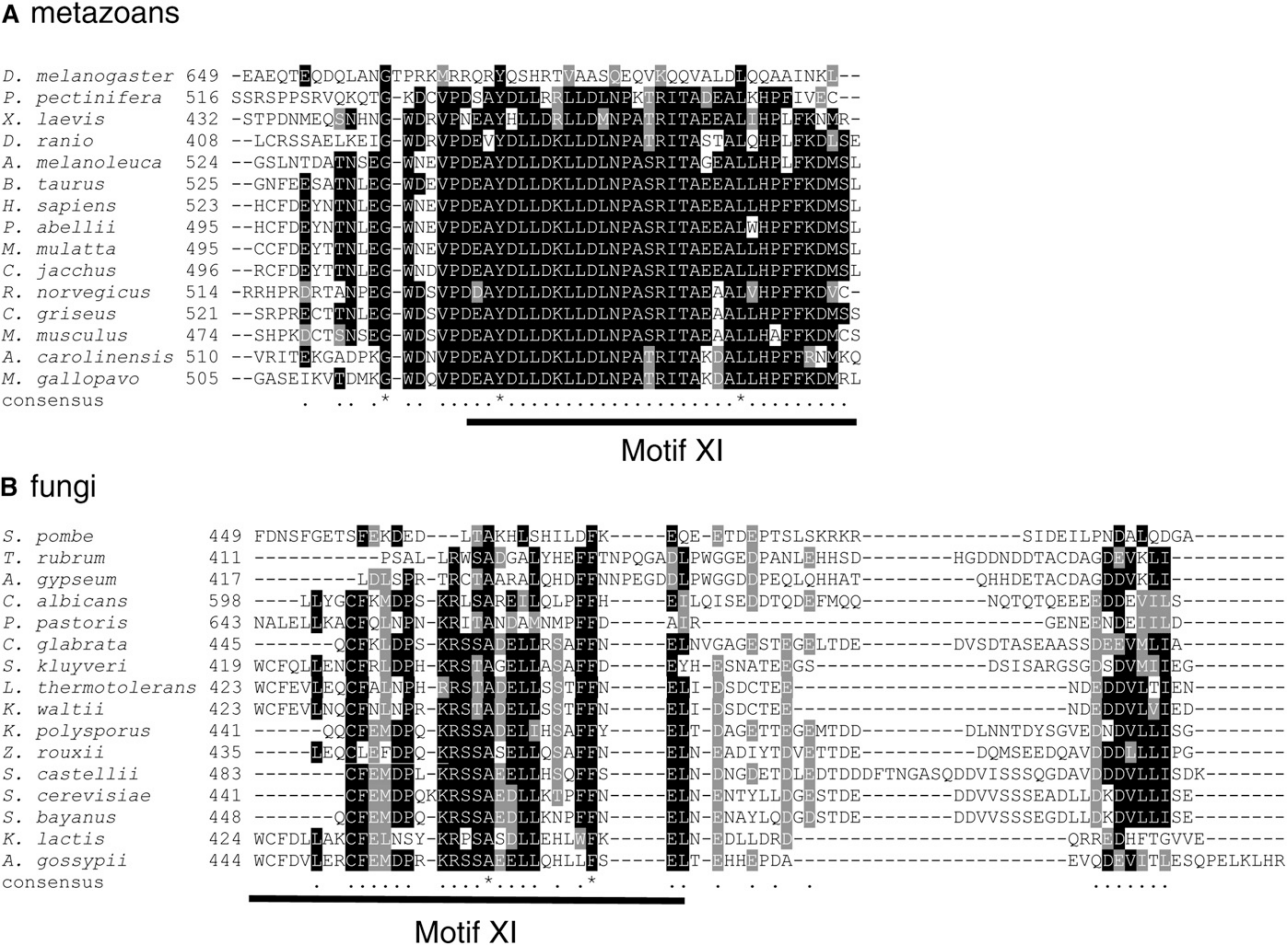
Alignment of Cdc7 C-terminal sequences. The C-terminal regions of Cdc7 orthologs from multicellular euakaryotes (A) and fungi (B) and were aligned using MUSCLE (Edgar 2004a, 2004b). Accession numbers of the full-length protein sequences are in Table S3. Black boxes indicate conserved residues and gray boxes similar residues in at least 50% of the sequences at the same position. The position of Motif XI is indicated for each alignment.

To examine whether the C-terminal residues of yeast and human Cdc7 are conserved through fungi and animals, we compared the ∼55 C-terminal residues from various organisms (Figure 8). The human sequence was strongly conserved among many metazoan species, particularly in the C-terminal proximal residues (residues 534-573 of human). Note that this region includes Motif XI of the eukaryotic kinase domain (Hanks *et al.* 1988; Hess *et al.* 1998). The strong similarity of the C-terminal region in multicellular eukaryotes suggests there is a selective pressure to maintain this sequence at this position of the protein. Many of these species also contain a second Dbf4 subunit (Drf1) or, in the case of mouse, two Dbf4 isoforms. Therefore, one pressure to maintain the C-terminal sequence may be its requirement to interact with two different subunits. Of note, the C terminus of the *D. melanogaster* Cdc7 protein (NP 727103) showed less similarity to human Cdc7 than the Cdc7 C-termini from other metazoans, even though *D. melanogaster* is predicted to encode two Dbf4 isoforms (NP 723965.1 and NP 523583.2). We speculate that this results from the fruit fly encoding a second Cdc7-like protein (NP 609876.2). The similarity among fungal species was not as strong as the similarity between most metazoan species. Interestingly, the strongest region of similarity within the fungal species corresponded to residues 441-523 of ScCdc7, which includes Motif XI and is consistent with the requirement of residues 449-561 for interaction with ScDbf4. With the exception of *S. pombe*, which encodes a second sporulation-specific Cdc7-Dbf4 complex (Nakamura *et al.* 2002), the fungal species contain only single Cdc7 and Dbf4 subunits. Our data suggest that Motif XI of Cdc7 encodes species-specific interaction with Dbf4.

### Implications for synthetic biology

To our knowledge, this is the first instance where complementation of a kinase deletion in yeast by the human ortholog requires the regulatory subunit. The complementation of a *S. pombe cdc2* deletion was famously used to clone human *cdc2*, a cyclin-dependent kinase, but it did not require expression of human cyclins to function (Lee and Nurse 1987). In addition, the human gene encoding the catalytic subunit of casein kinase II (CK2α) complements deletion of the *S. cerevisiae* genes (there are two different catalytic subunits in budding yeast) without need of the regulatory subunit (Dotan *et al.* 2001). With DDK, the requirement of the regulatory subunit to achieve complementation serves as a guide for creation of hybrid synthetic genomes where consideration of the structure/function of multicomponent enzymes will be required. Of note, the component genes of DDK are found on different arms of the same chromosome (IV) in *S. cerevisiae* but on different chromosomes (1 and 7) in humans.

The species specificity of Cdc7-Dbf4 interaction is a potential area to exploit for the development of anti-mycotic drugs. Additionally, this genetically amenable system can be used to rapidly probe the key structure/function relationships of HsCdc7 and its interactions with potential cancer therapeutics. Human DDK in yeast may also prove useful for study of DDK’s nonessential roles in DNA-damage response and meiosis.

## Supplementary Material

Supporting Information
