# Supplementary material for: A Synthetic Human Kinase Can Control Cell Cycle Progression in Budding Yeast
Source: G3 (Bethesda). 2011 Sep 1;1(4):317–25. doi: 10.1534/g3.111.000430 (PMC3276143; doi:10.1534/g3.111.000430)
Supplement: Supporting Information [file supp_1_4_317__index.html]

Supporting Information 

# A Synthetic Human Kinase Can Control Cell Cycle Progression in Budding Yeast

## Supporting Information for Davey *et al.*, 2011

**Files in this Data Supplement:**

- Supporting Information - Figure S1-S5 and Table S1-S3 (PDF, 884 KB)
- Figure S1 - Co-IP to test interaction of human and yeast proteins (PDF, 116 KB)
- Figure S2 - PCR analysis of strains containing human CDC7 and DBF4 (PDF, 172 KB)
- Figure S3 - Growth curves of yDDk and hDDk strains (PDF, 100 KB)
- Figure S4 - Expression of myc-tagged human Cc7, Dbf4 and Drf1 (PDF, 200 KB)
- Figure S5 - Western blotting of myc9-tagged Cdc7 hybrid proteins (PDF, 140 KB)
- Table S1 - Oligonucleotides used in this study (PDF, 44 KB)
- Table S2 - Yeast strains used in this study (PDF, 108 KB)
- Table S3 - Accession numbers of Cdc7 sequences in Figure 8 (PDF, 48 KB)
