# Supplementary material for: A Synthetic Human Kinase Can Control Cell Cycle Progression in Budding Yeast
Source: G3 (Bethesda). 2011 Sep 1;1(4):317–25. doi: 10.1534/g3.111.000430 (PMC3276143; doi:10.1534/g3.111.000430)
Supplement: Supporting Information [file supp_1.4.317_000430.pdf]

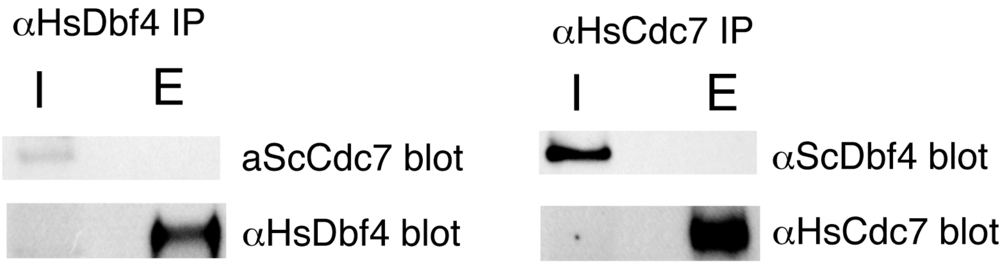

Figure S1 Co-IP to test interaction of human and yeast proteins. Protein extracts were made from CY4242 and then treated with Protein G Dynabeads and the indicated antibody (top panel). After washing and elution from the beads, 50% of the elution ("E") and approximately 1% of the input ("I") were separated by SDS-PAGE, transferred to PVDF and then probed with the indicated antibodies.

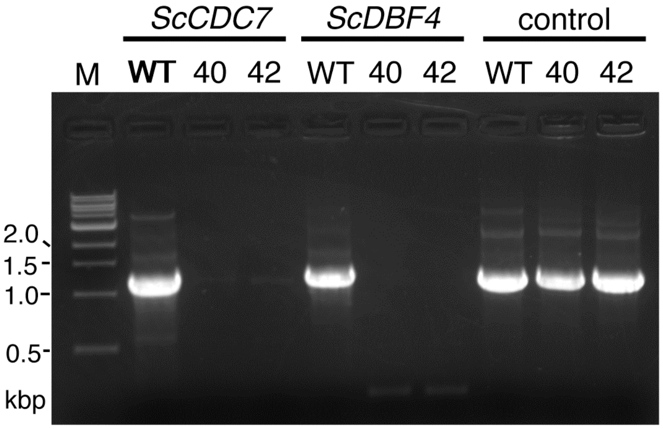

Figure S2 PCR analysis of strains containing human CDC7 and DBF4: Genomic DNA was prepared from wild type yeast (BY4741; "WT"), and strains maintained by *HsCDC7* and *HsDBF4* (CY4240; "40" and CY4242 "42") and then probed for the presence of *ScCDC7* or *ScDBF4* by PCR using the primers in Table S1. Shown is an ethidium bromide stained 0.8% agorose gel with a portion of each sample. The control product is *ScMCM7*, present in all strains.

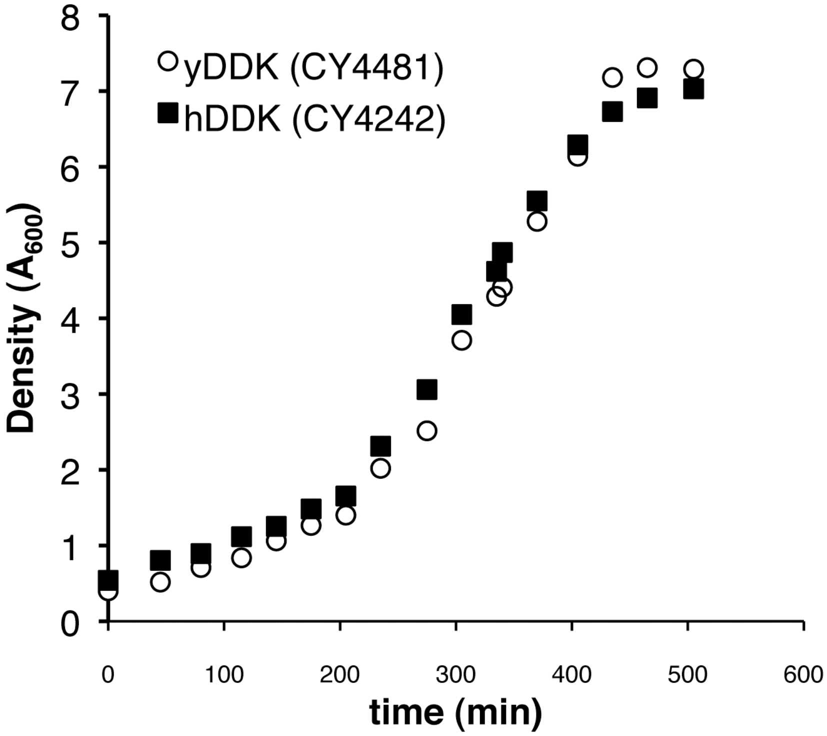

Figure S3 Growth curves of yDDk and hDDk strains. CY4481 and CY424 were grown overnight in YPD and then diluted to an A_{600} of approximately 0.05 in YPD. The cultures were incubated at 30° with shaking. At the indicated times, an aliquot was removed and the A_{600} measured.

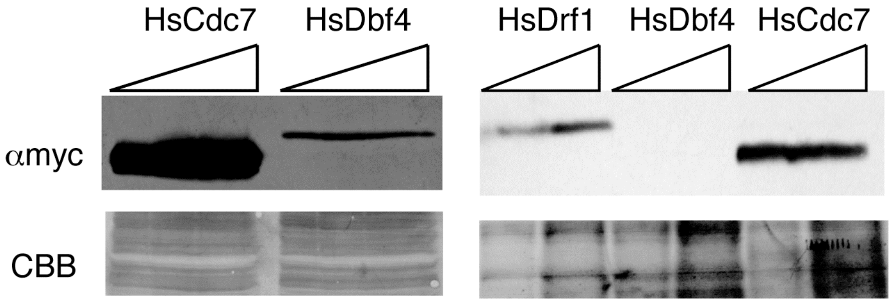

Figure S4 Expression of myc-tagged human Cc7, Dbf4 and Drf1: The strain BY4742 was transformed with plasmids expressing myc[9] –HsCdc7, myc[9] –HsDbf4 and myc[9] –HsDrf1, grown overnight at 30° in selective media and lyzed using NaOH and SDS as described (AMBERG *et al*., 2005). Lysates were subjected to electrophoresis by SDS-PAGE (8%) and then transferred to PVDF. The membranes were probed with anti-myc antibody as described in the Methods and Materials of the main text. The upper panel shows the signal from antibody (αmyc) and the lower panel Coomassie Brilliant Blue R250 staining of the membrane (CBB). Since HsDbf4 was not visualized in the experiment that included HsDrf1, we concluded that HsDrf1 is expressed at least as well as HsDbf4.

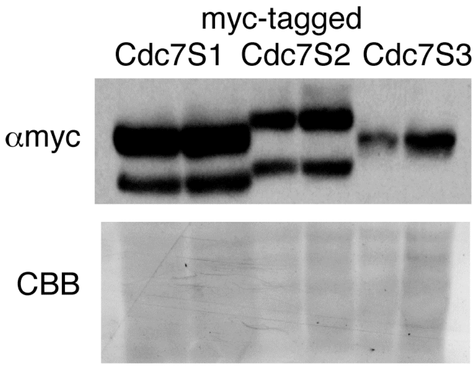

Figure S5 Western blotting of myc^9-tagged Cdc7 hybrid proteins. Whole cell lysates of BY4741 transformed with myc^9-Cdc7S1, myc^9-Cdc7S2 or myc^9-Cdc7S3 were separated by SDS-PAGE, transferred to nitrocellulose and probed with anti-myc antibody. The top panel shows the Western blot and the lower panel is the membrane after staining with Coomassie Brilliant Blue R250 stain.

Table S1 Oligonucleotides used in this study

Name	Purpose	Sequence
MD405	*ScCDC7* promoter	GGAATTCCCCGGGGACGGAGTTTTTTAGTCAGTTC
MD406		GGAATTCGTCGACCATATGTATGATTGTTCTACTTCGAAACTG
MD387	*ScDBF4* ORF +	GGAATTCGTCGACAAAAGAACAGTAAGAAAGAAGAC
MD390	promoter	GGAATTCCCTGCAGGCAATAACATTGCCGTTGATAGC
5556-1	*HsCDC7* ORF *Nde*I/*Sbf*I	GGGAATTCCATATGGAGGCGTCTTTGGGGATT
5556-2		GGAATTCCCTGCAGGTACCTCATAACAGTAAACATTAAATG
5556-3	HsDBF4 ORF *Nde*I/*Sbf*I	GGGAATTCCATATGAACTCCGGAGCCATGAG
5556-4		GGAATTCCCTGCAGGATCCTTATCACTTCTGAAAAGT
MD519	*HsCDC7* myc plasmid	ATGACGCGGCCGCGATGGAGGCGTCTTTGGGGATTC
MD520		TAGGCTGAGCTCTACCTCATAACAGTAAACATTAAATG
MD521	*HsDBF4* myc plasmid	ATGACGCGGCCGCCATGAACTCCGGAGCCATGAG
MD522		TAGGCTGAGCTCATCCTTATCACTTCTGAAAAGT
MD235	*ScCDC7* detection	AATGAGCTCAACCTGCTG
MD237		GAGAACATCCTTATCGAGC
MD238	*ScDBF4* detection	TCCAAGTCAGCAAAGGTAC
MD240		ATTGCCAAAAGAGGTTGC
MD451	control (*MCM7*)	CTGCTGAAGGCCATTTGC
MD452		GATCAGGCTCAGCAGAATGAAGGCCCTGTTGC
MD508	*HsDRF1* ORF	GATACCATATGAGCGAACCGGGAAAGGGAG
MD509		ATGCATGGATCCTTCAGGTGTCTCGGAGCTGAC
MD538	*HsCDC7 Xba*I	GGACTTTCTAGACAGACTAGCAGTAATTTATCAC
MD564	hybrid 1 overlap	GTGAGCATTGTTTCGAAATGGATCCTCAAAAGC
MD566	primers	ATCCATTTCGAAAGAATGCTCACAGCTATTACTATC
MD577	hybrid 2 overlap	AAACGGGGTTGGTACCTCATTCCAGCCTT
MD578	primers	ATGAGGTACCAACCCCGTTTTTCAATGAATTGC
MD572	hybrid 3 overlap	AACGTCATCTTTGAAGCTGGATTTAGATCTAG
MD573	primers	CCAGCTTCAAAGATGACGTTGTCAGCTCAAGC
MD567	*ScCDC7 Sbf*I	GGAATTCCCTGCAGGGCTATTCAGATATTAGGAGAAC
MD593	*HsDRF1 Not*I	ATGACGCGGCCGCGATGAGCGAACCGGGAAAGGGAG
MD594	*HsDRF1 Eco*RI	GATCAGGAATTCTTCAGGTGTCTCGGAGCTGAC

Table S2 Yeast strains used in this study

Name	DDK alleles	Relevant genotype	Source
BY4741	*CDC7 DBF4*	*MATa his3Δ1 leu2Δ0 met15Δ0 ura3Δ0*	(GIAEVER *et al.* 2002)
BY23713	*CDC7/cdc7Δ*	*MATa/α his3Δ1 leu2Δ0 LYS2/lys2Δ0 MET15/met15Δ0 ura3Δ0/ura3Δ0 CDC7/cdc7::KanMX*	(GIAEVER *et al.* 2002)
BY23988	*DBF4/dbf4Δ*	*MATa/α his3Δ1 leu2Δ0 LYS2/lys2Δ0 MET15/met15Δ0 ura3Δ0/ura3Δ0 DBF4/dbf4::KanMX*	(GIAEVER *et al.* 2002)
MDY95	*cdc7Δ ScCDC7-URA3*	*MATa his3Δ1 leu2Δ0 ura3Δ0 cdc7::KanMX* YCplac33-*ScCDC7*	This study
CY4104	*dbf4Δ ScDBF4-URA3*	*MATα his3Δ1 leu2Δ0 ura3Δ0 dbf4::KanMX* YCplac33-*ScDBF4*	This study
CY4178	*dbf4Δ ScDBF4-URA3*	*MATα his3Δ1 leu2Δ0 ura3Δ0 dbf4::NatMX* YCplac33-*ScDBF4*	This study
CY4348	*CDC7/cdc7Δ DBF4/dbf4Δ*	*MATa/α his3Δ1/his3Δ1 leu2Δ0/leu2Δ0 ura3Δ0/ura3Δ0 CDC7/cdc7::KanMX/ DBF4/dbf4::NatMX*	This study
MDY195	*cdc7Δ ScCDC7-LEU2*	*MATa his3Δ1 leu2Δ0 ura3Δ0 cdc7::KanMX* YCplac111-*ScCDC7*	This study
CY5535	*cdc7Δ ScCDC7-URA3 ScCDC7-LEU2*	*MATa his3Δ1 leu2Δ0 ura3Δ0 cdc7::KanMX* YCplac33-*ScCDC7* YCplac111-*ScCDC7*	
CY4328	*dbf4Δ ScDBF4-URA3 HsCDC7-CEN HsDBF4-CEN*	CY4104 YCplac111h-*HsCDC7* YCplac111-*HsDBF4*	This study
CY4505	*dbf4Δ ScDBF4-URA3 HsCDC7-2 μ HsDBF4-CEN*	CY4104 YEplac181h-*HsCDC7* YCplac111-*HsDBF4*	This study
CY4343	*dbf4Δ ScDBF4-URA3 HsCDC7-CEN HsDBF4-2μ*	CY4104 YCplac111h-*HsCDC7* YEplac181-*HsDBF4*	This study
CY4344	*dbf4Δ ScDBF4-URA3 HsCDC7-2 μ HsDBF4-2μ*	CY4104 YEplac181h-*HsCDC7* YEplac181-*HsDBF4*	This study
CY4176	*dbf4Δ HsCDC7-CEN HsDBF4-2μ*	*MATα his3Δ1 leu2Δ0 ura3Δ0 dbf4::KanMX* YCplac111h-*HsCDC7* YEplac181-*HsDBF4*	This study
CY4177	*dbf4Δ HsCDC7-2 μ HsDBF4-2μ*	*MATα his3Δ1 leu2Δ0 ura3Δ0 dbf4::NatMX* YEplac181h-*HsCDC7* YEplac181-*HsDBF4*	This study
CY4500	*cdc7Δ ScCDC7-URA3 HsCDC7-CEN HsDBF4-2μ*	MDY95 *YCplac111h-HsCDC7* YEplac181-*HsDBF4*	This study
CY4501	*cdc7Δ ScCDC7-URA3 HsCDC7-2 μ HsDBF4-2μ*	MDY95 YEplac181h-*HsCDC7* YEplac181-*HsDBF4*	This study
CY4245	*cdc7Δ HsCDC7-CEN HsDBF4-2μ*	*MATa his3Δ1 leu2Δ0 ura3Δ0 cdc7::KanMX* YCplac111h-*HsCDC7* YEplac181-*HsDBF4*	This study
CY4246	*cdc7Δ ScCDC7-URA3 HsCDC7-2 μ HsDBF4-2μ*	*MATa his3Δ1 leu2Δ0 ura3Δ0 cdc7::KanMX* YEplac181h-*HsCDC7* YEplac181-*HsDBF4*	This study
CY4481	*cdc7Δ dbf4Δ ScCDC7-LEU2 ScDBF4-URA3*	*MATa his3Δ1 leu2Δ0 ura3Δ0 cdc7::KanMX dbf4::NatMX* YCplac111-*ScCDC7* YCplac33-*ScDBF4*	This study
CY4240	*cdc7Δ dbf4Δ HsCDC7-CEN*	*MATα his3Δ1 leu2Δ0 ura3Δ0 cdc7::KanMX dbf4::NatMX* YCplac111h-*HsCDC7* YEplac181-*HsDBF4*	This study

	HsDBF4-2µ		
CY4242	*cdc7Δ dbf4Δ HsCDC7-2 µ HsDBF4-2µ*	*MATα his3Δ1 leu2Δ0 ura3Δ0 cdc7::KanMX dbf4::NatMX* YEplac181h-*HsCDC7* YEplac181-*HsDBF4*	This study
MDY214	*cdc7Δ dbf4Δ ScCDC7-ScDBF4-URA3*	*MATα his3Δ1 leu2Δ0 ura3Δ0 cdc7::KanMX dbf4::NatMX* YCplac33-*ScCdc7-ScDBF4*	This study
CY4507	*cdc7Δ dbf4Δ ScCDC7-ScDBF4-URA3 HsDBF4 2µ HsCDC7 CEN*	*MATα his3Δ1 leu2Δ0 ura3Δ0 cdc7::KanMX dbf4::NatMX* YCplac33-*ScCdc7-ScDBF4* YEplac181-*HsDBF4* YCplac111h-*HsCDC7*	This study
CY4508	*cdc7Δ dbf4Δ ScCDC7-ScDBF4-URA3 HsDBF4 2 µ HsCDC7-2µ*	*MATα his3Δ1 leu2Δ0 ura3Δ0 cdc7::KanMX dbf4::NatMX* YCplac33-*ScCdc7-ScDBF4* YEplac181-*HsDBF4* YEplac181h-*HsCDC7*	This study
CY5536	*cdc7Δ dbf4Δ ScCDC7-ScDBF4-URA3 HsDRF1-CEN HsCDC7 CEN*	*MATα his3Δ1 leu2Δ0 ura3Δ0 cdc7::KanMX dbf4::NatMX* YCplac33-*ScCdc7-ScDBF4* YCplac111-*HsDRF1* YCplac111h-*HsCDC7*	This study
CY5537	*cdc7Δ dbf4Δ ScCDC7-ScDBF4-URA3 HsDRF1-CEN HsCDC7 2 µ*	*MATα his3Δ1 leu2Δ0 ura3Δ0 cdc7::KanMX dbf4::NatMX* YCplac33-*ScCdc7-ScDBF4* YCplac111-*HsDRF1* YEplac181h-*HsCDC7*	This study
CY5538	*cdc7Δ dbf4Δ ScCDC7-ScDBF4-URA3 HsDRF1-2 µ HsCDC7 CEN*	*MATα his3Δ1 leu2Δ0 ura3Δ0 cdc7::KanMX dbf4::NatMX* YCplac33-*ScCdc7-ScDBF4* YEplac181-*HsDRF1* YCplac111h-*HsCDC7*	This study
CY5539	*cdc7Δ dbf4Δ ScCDC7-ScDBF4-URA3 HsDRF1-2 µ HsCDC7 2 µ*	*MATα his3Δ1 leu2Δ0 ura3Δ0 cdc7::KanMX dbf4::NatMX* YCplac33-*ScCdc7-ScDBF4* YEplac181-*HsDRF1* YEplac181h-*HsCDC7*	This study
CY5628	*cdc7Δ/cdc7Δ dbf4Δ/dbf4Δ HsCDC7-CEN HsDBF4-2 µ*	*MATa/α his3Δ1/his3Δ1 leu2Δ0/leu2Δ0 ura3Δ0/ura3Δ0 cdc7::KanMX/cdc7::KanMX dbf4::NatM/dbf4::NatMX* YCplac111h-*HsCDC7* YCplac181-*HsDBF4*	This study
CY5627	*cdc7Δ/cdc7Δ dbf4Δ/dbf4Δ ScCDC7-ScDBF4-URA3*	*MATa/α his3Δ1/his3Δ1 leu2Δ0/leu2Δ0 ura3Δ0/ura3Δ0 cdc7::KanMX/cdc7::KanMX dbf4::NatM/dbf4::NatMX* YCplac33-*ScCdc7-ScDBF4*	This study
MDY270	*cdc7Δ/cdc7Δ dbf4Δ/dbf4Δ ScCDC7-ScDBF4-URA3 HsCDC7-CEN HsDBF4-2 µ*	*MATa/α his3Δ1/his3Δ1 leu2Δ0/leu2Δ0 ura3Δ0/ura3Δ0 cdc7::KanMX/cdc7::KanMX dbf4::NatM/dbf4::NatMX YCplac33-ScCdc7-ScDBF4* YCplac111h-*HsCDC7* YCplac181-*HsDBF4*	This study
YSC1178-7499555	TAP-Cdc7	*MATa his3Δ1 leu2Δ0 ura3Δ0 CDC7-TAP HIS3*	(GHAEMMAGHAMI *et al.* 2003)
MDY201	TAP-Cdc7 myc^{9}-HsDbf4	*MATa his3Δ1 leu2Δ0 ura3Δ0 CDC7-TAP HIS3* YCp88-*myc*^{9}-*HsDBF4*	This study
YSC1178-7499753	TAP-Dbf4	*MATa his3Δ1 leu2Δ0 ura3Δ0 DBF4-TAP HIS3*	(GHAEMMAGHAMI *et al.* 2003)
MDY200	TAP-Dbf4 myc^{9}-HsCdc7	*MATa his3Δ1 leu2Δ0 ura3Δ0 DBF4-TAP HIS3* YCp88-*myc*^{9}-*HsCDC7*	This study

MDY265	*cdc7Δ ScCDC7-URA3 CDC7-S1*	*MATa his3Δ1 leu2Δ0 ura3Δ0 cdc7::KanMX* YCplac33-*ScCDC7 YEplac181h CDC7-S1*	This study
MDY266	*cdc7Δ CDC7-S1*	*MATa his3Δ1 leu2Δ0 ura3Δ0 cdc7::KanMX* YEplac181-*CDC7-S1*	This study
MDY267	*cdc7Δ ScCDC7-URA3 CDC7-S2*	*MATa his3Δ1 leu2Δ0 ura3Δ0 cdc7::KanMX* YCplac33-*ScCDC7* YEplac181-*CDC7-S2*	This study
MDY268	*cdc7Δ ScCDC7-URA3 CDC7-S3*	*MATa his3Δ1 leu2Δ0 ura3Δ0 cdc7::KanMX* YCplac33-*ScCDC7* YEplac181-*CDC7-S3*	This study
MDY269	*dbf4Δ ScDBF4-URA3 CDC7-S1 HsDBF4 2 μ*	*MATα his3Δ1 leu2Δ0 ura3Δ0 dbf4::KanMX* YCplac33-*ScDBF4* YEplac181h-*CDC7-S1* YEplac181-*HsDBF4*	This study
MDY317	*cdc7Δ/cdc7Δ dbf4Δ/dbf4Δ ScCDC7-ScDBF4-URA3 CDC7-S1 ScDBF4-LEU2*	*MATa/α his3Δ1/his3Δ1 leu2Δ0/leu2Δ0 ura3Δ0/ura3Δ0 cdc7::KanMX/cdc7::KanMX dbf4::NatM/dbf4::NatMX* YCplac33-*ScCdc7-ScDBF4* YCplac111h-*CDC7-S1* YCplac33L-*ScDBF4*	This study
MDY318	*cdc7Δ/cdc7Δ dbf4Δ/dbf4Δ CDC7-S1 ScDBF4-LEU2*	*MATa/α his3Δ1/his3Δ1 leu2Δ0/leu2Δ0 ura3Δ0/ura3Δ0 cdc7::KanMX/cdc7::KanMX dbf4::NatM/dbf4::NatMX* YCplac111h-*CDC7-S1* YCplac33L-*ScDBF4*	This study

Table S3 Accession numbers of Cdc7 sequences in Figure 8

Species Name	Accession Number
Drosophila melanogaster	NP_727103
Patiria pectinifera	BAI68101.1
Xenopus laevis	AAD21532.1
Danio rerio	NP_001007410.1
Ailuropoda melanoleuca	XP_002926239.1
Bos taurus	DAA31396.1
Homo sapiens	AAC52080.1
Pongo abelii	XP_002810658.1
Macaca mulatta	XP_002801693.1
Callithrix jacchus	XP_002801693.1
Rattus norvegicus	NP_001101822.1
Cricetulus griseus	AAK29327.1
Mus musculus	AAH80702.1
Anolis carolensis	XP_003220149.1
Meleagris gallopavo	XP_003208745.1
Schizosaccharomyces pombe	NP_596328.1
Trichophyton rubrum	XP_003237969.1
Arthroderma gypseum	XP_003173222.1
Candida albicans	AAW28083.1
Pichia pastoris	CCA37102.1
Candida glabrata	XP_448304.1
Saccharomyces kluyveri	SAKL0C07876g
Lachancea thermotolerans	XP_002554175.1
Kluyveromyces waltii	Kwal_27.9804 s27
Vanderwaltozyma polyspora	XP_001645808.1
Zygosaccharomyces rouxii	XP_002494590.1
Saccharomyces castellii	Scas_718.72 s2003
Saccharomyces cerevisiae	NP_010267.1
Saccharomyces bayanus	Sbay_542.8 c542
Kluyveromyces lactis	XP_452391.1
Ashbya gossypii	NP_985073.1
