# Supplementary material for: A Synthetic Human Kinase Can Control Cell Cycle Progression in Budding Yeast
Source: G3 (Bethesda). 2011 Sep 1;1(4):317–25. doi: 10.1534/g3.111.000430 (PMC3276143; doi:10.1534/g3.111.000430)
Supplement: Supporting Information [file supp_1.4.317_FigureS1.pdf]

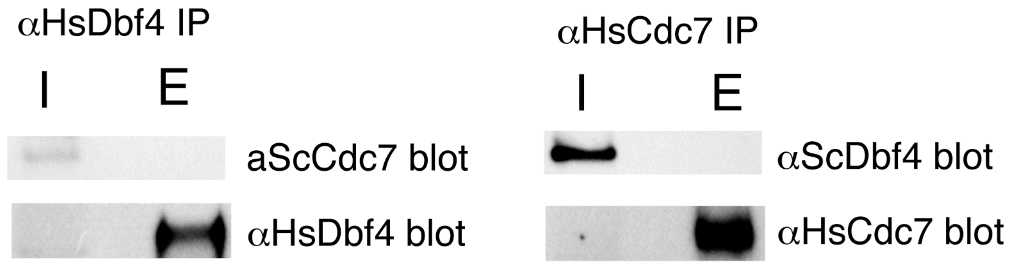


**Figure S1** Co-IP to test interaction of human and yeast proteins. Protein extracts were made from CY4242 and then treated with Protein G Dynabeads and the indicated antibody (top panel). After washing and elution from the beads, 50% of the elution ("E") and approximately 1% of the input ("I") were separated by SDS-PAGE, transferred to PVDF and then probed with the indicated antibodies.
