# Supplementary material for: A Synthetic Human Kinase Can Control Cell Cycle Progression in Budding Yeast
Source: G3 (Bethesda). 2011 Sep 1;1(4):317–25. doi: 10.1534/g3.111.000430 (PMC3276143; doi:10.1534/g3.111.000430)
Supplement: Supporting Information [file supp_1.4.317_FigureS2.pdf]

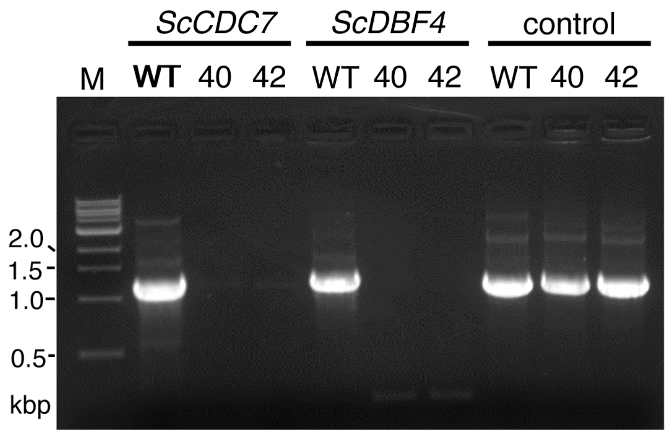

Figure S2 PCR analysis of strains containing human CDC7 and DBF4: Genomic DNA was prepared from wild type yeast (BY4741; “WT”), and strains maintained by *HsCDC7* and *HsDBF4* (CY4240; “40” and CY4242 “42”) and then probed for the presence of *ScCDC7* or *ScDBF4* by PCR using the primers in Table S1. Shown is an ethidium bromide stained 0.8% agorose gel with a portion of each sample. The control product is *ScMCM7*, present in all strains.
