# Supplementary material for: A Synthetic Human Kinase Can Control Cell Cycle Progression in Budding Yeast
Source: G3 (Bethesda). 2011 Sep 1;1(4):317–25. doi: 10.1534/g3.111.000430 (PMC3276143; doi:10.1534/g3.111.000430)
Supplement: Supporting Information [file supp_1.4.317_FigureS3.pdf]

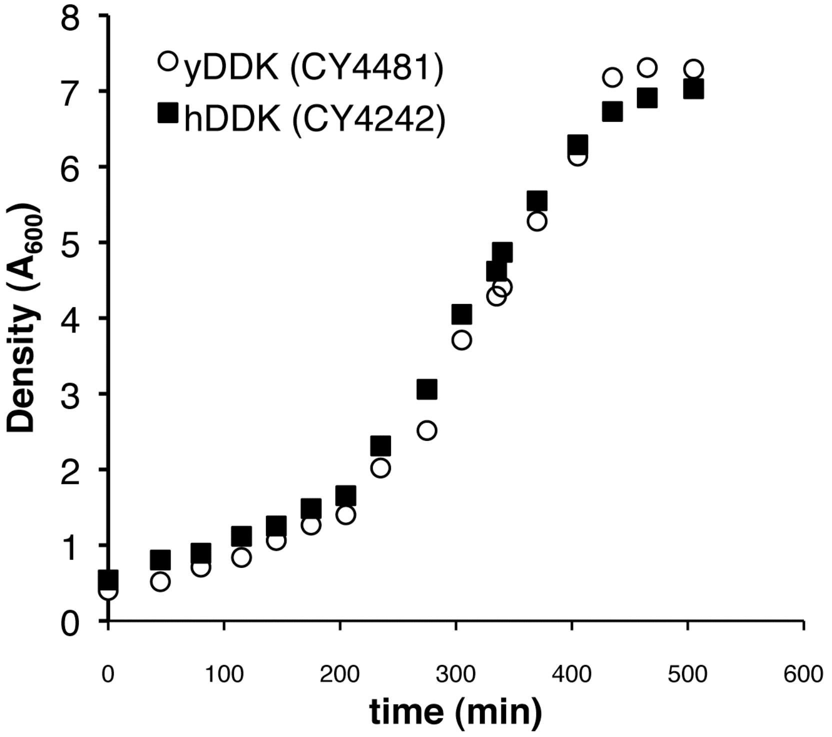

Figure S3 Growth curves of yDDk and hDDk strains. CY4481 and CY424 were grown overnight in YPD and then diluted to an A_{600} of approximately 0.05 in YPD. The cultures were incubated at 30° with shaking. At the indicated times, an aliquot was removed and the A_{600} measured.
