# Supplementary material for: A Synthetic Human Kinase Can Control Cell Cycle Progression in Budding Yeast
Source: G3 (Bethesda). 2011 Sep 1;1(4):317–25. doi: 10.1534/g3.111.000430 (PMC3276143; doi:10.1534/g3.111.000430)
Supplement: Supporting Information [file supp_1.4.317_FigureS4.pdf]

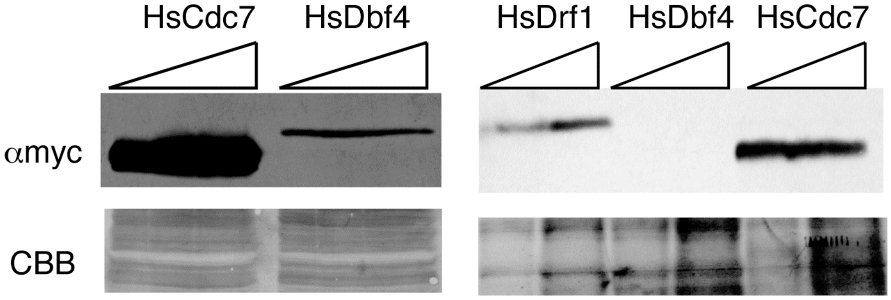

Figure S4 Expression of myc-tagged human Cc7, Dbf4 and Drf1: The strain BY4742 was transformed with plasmids expressing myc^9 –HsCdc7, myc^9 –HsDbf4 and myc^9 –HsDrf1, grown overnight at 30° in selective media and lyzed using NaOH and SDS as described (Amberg *et al*., 2005). Lysates were subjected to electrophoresis by SDS-PAGE (8%) and then transferred to PVDF. The membranes were probed with anti-myc antibody as described in the Methods and Materials of the main text. The upper panel shows the signal from antibody (αmyc) and the lower panel Coomassie Brilliant Blue R250 staining of the membrane (CBB). Since HsDbf4 was not visualized in the experiment that included HsDrf1, we concluded that HsDrf1 is expressed at least as well as HsDbf4.
