# Supplementary material for: A Synthetic Human Kinase Can Control Cell Cycle Progression in Budding Yeast
Source: G3 (Bethesda). 2011 Sep 1;1(4):317–25. doi: 10.1534/g3.111.000430 (PMC3276143; doi:10.1534/g3.111.000430)
Supplement: Supporting Information [file supp_1.4.317_FigureS5.pdf]

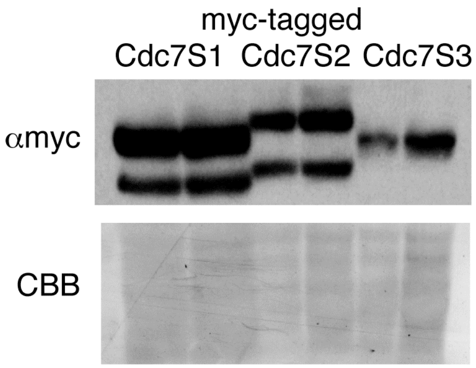

Figure S5 Western blotting of myc^9-tagged Cdc7 hybrid proteins. Whole cell lysates of BY4741 transformed with myc^9-Cdc7S1, myc^9-Cdc7S2 or myc^9-Cdc7S3 were separated by SDS-PAGE, transferred to nitrocellulose and probed with anti-myc antibody. The top panel shows the Western blot and the lower panel is the membrane after staining with Coomassie Brilliant Blue R250 stain.
