# Supplementary material for: A Synthetic Human Kinase Can Control Cell Cycle Progression in Budding Yeast
Source: G3 (Bethesda). 2011 Sep 1;1(4):317–25. doi: 10.1534/g3.111.000430 (PMC3276143; doi:10.1534/g3.111.000430)
Supplement: Supporting Information [file supp_1.4.317_TableS1.pdf]

Table S1 Oligonucleotides used in this study

Name	Purpose	Sequence
MD405	*ScCDC7* promoter	GGAATTCCCCGGGGACGGAGTTTTTTAGTCAGTTC
MD406		GGAATTCGTCGACCATATGTATGATTGTTCTACTTCGAAACTG
MD387	*ScDBF4* ORF +	GGAATTCGTCGACAAAAGAACAGTAAGAAAGAAGAC
MD390	promoter	GGAATTCCCTGCAGGCAATAACATTGCCGTTGATAGC
5556-1	*HsCDC7* ORF *Nde*I/*Sbf*I	GGGAATTCCATATGGAGGCGTCTTTGGGGATT
5556-2		GGAATTCCCTGCAGGTACCTCATAACAGTAAACATTAAATG
5556-3	HsDBF4 ORF *Nde*I/*Sbf*I	GGGAATTCCATATGAACTCCGGAGCCATGAG
5556-4		GGAATTCCCTGCAGGATCCTTATCACTTCTGAAAAGT
MD519	*HsCDC7* myc plasmid	ATGACGCGGCCGCGATGGAGGCGTCTTTGGGGATTC
MD520		TAGGCTGAGCTCTACCTCATAACAGTAAACATTAAATG
MD521	*HsDBF4* myc plasmid	ATGACGCGGCCGCCATGAACTCCGGAGCCATGAG
MD522		TAGGCTGAGCTCATCCTTATCACTTCTGAAAAGT
MD235	*ScCDC7* detection	AATGAGCTCAACCTGCTG
MD237		GAGAACATCCTTATCGAGC
MD238	*ScDBF4* detection	TCCAAGTCAGCAAAGGTAC
MD240		ATTGCCAAAAGAGGTTGC
MD451	control (*MCM7*)	CTGCTGAAGGCCATTTGC
MD452		GATCAGGCTCAGCAGAATGAAGGCCCTGTTGC
MD508	*HsDRF1* ORF	GATACCATATGAGCGAACCGGGAAAGGGAG
MD509		ATGCATGGATCCTTCAGGTGTCTCGGAGCTGAC
MD538	*HsCDC7 Xba*I	GGACTTTCTAGACAGACTAGCAGTAATTTATCAC
MD564	hybrid 1 overlap	GTGAGCATTGTTTCGAAATGGATCCTCAAAAGC
MD566	primers	ATCCATTTCGAAAGAATGCTCACAGCTATTACTATC
MD577	hybrid 2 overlap	AAACGGGGTTGGTACCTCATTCCAGCCTT
MD578	primers	ATGAGGTACCAACCCCGTTTTTCAATGAATTGC
MD572	hybrid 3 overlap	AACGTCATCTTTGAAGCTGGATTTAGATCTAG
MD573	primers	CCAGCTTCAAAGATGACGTTGTCAGCTCAAGC
MD567	*ScCDC7 Sbf*I	GGAATTCCCTGCAGGGCTATTCAGATATTAGGAGAAC
MD593	*HsDRF1 Not*I	ATGACGCGGCCGCGATGAGCGAACCGGGAAAGGGAG
MD594	*HsDRF1 Eco*RI	GATCAGGAATTCTTCAGGTGTCTCGGAGCTGAC
