# Supplementary material for: A Synthetic Human Kinase Can Control Cell Cycle Progression in Budding Yeast
Source: G3 (Bethesda). 2011 Sep 1;1(4):317–25. doi: 10.1534/g3.111.000430 (PMC3276143; doi:10.1534/g3.111.000430)
Supplement: Supporting Information [file supp_1.4.317_TableS2.pdf]

Table S2 Yeast strains used in this study

Name	DDK alleles	Relevant genotype	Source
BY4741	*CDC7 DBF4*	*MATa his3Δ1 leu2Δ0 met15Δ0 ura3Δ0*	(GIAEVER *et al.* 2002)
BY23713	*CDC7/cdc7Δ*	*MATa/α his3Δ1 leu2Δ0 LYS2/lys2Δ0 MET15/met15Δ0 ura3Δ0/ura3Δ0 CDC7/cdc7::KanMX*	(GIAEVER *et al.* 2002)
BY23988	*DBF4/dbf4Δ*	*MATa/α his3Δ1 leu2Δ0 LYS2/lys2Δ0 MET15/met15Δ0 ura3Δ0/ura3Δ0 DBF4/dbf4::KanMX*	(GIAEVER *et al.* 2002)
MDY95	*cdc7Δ ScCDC7-URA3*	*MATa his3Δ1 leu2Δ0 ura3Δ0 cdc7::KanMX* YCplac33-*ScCDC7*	This study
CY4104	*dbf4Δ ScDBF4-URA3*	*MATα his3Δ1 leu2Δ0 ura3Δ0 dbf4::KanMX* YCplac33-*ScDBF4*	This study
CY4178	*dbf4Δ ScDBF4-URA3*	*MATα his3Δ1 leu2Δ0 ura3Δ0 dbf4::NatMX* YCplac33-*ScDBF4*	This study
CY4348	*CDC7/cdc7Δ DBF4/dbf4Δ*	*MATa/α his3Δ1/his3Δ1 leu2Δ0/leu2Δ0 ura3Δ0/ura3Δ0 CDC7/cdc7::KanMX/ DBF4/dbf4::NatMX*	This study
MDY195	*cdc7Δ ScCDC7-LEU2*	*MATa his3Δ1 leu2Δ0 ura3Δ0 cdc7::KanMX* YCplac111-*ScCDC7*	This study
CY5535	*cdc7Δ ScCDC7-URA3 ScCDC7-LEU2*	*MATa his3Δ1 leu2Δ0 ura3Δ0 cdc7::KanMX* YCplac33-*ScCDC7* YCplac111-*ScCDC7*	
CY4328	*dbf4Δ ScDBF4-URA3 HsCDC7-CEN HsDBF4-CEN*	CY4104 YCplac111h-*HsCDC7* YCplac111-*HsDBF4*	This study
CY4505	*dbf4Δ ScDBF4-URA3 HsCDC7-2 μ HsDBF4-CEN*	CY4104 YEplac181h-*HsCDC7* YCplac111-*HsDBF4*	This study
CY4343	*dbf4Δ ScDBF4-URA3 HsCDC7-CEN HsDBF4-2μ*	CY4104 YCplac111h-*HsCDC7* YEplac181-*HsDBF4*	This study
CY4344	*dbf4Δ ScDBF4-URA3 HsCDC7-2 μ HsDBF4-2μ*	CY4104 YEplac181h-*HsCDC7* YEplac181-*HsDBF4*	This study
CY4176	*dbf4Δ HsCDC7-CEN HsDBF4-2μ*	*MATα his3Δ1 leu2Δ0 ura3Δ0 dbf4::KanMX* YCplac111h-*HsCDC7* YEplac181-*HsDBF4*	This study
CY4177	*dbf4Δ HsCDC7-2 μ HsDBF4-2μ*	*MATα his3Δ1 leu2Δ0 ura3Δ0 dbf4::NatMX* YEplac181h-*HsCDC7* YEplac181-*HsDBF4*	This study
CY4500	*cdc7Δ ScCDC7-URA3 HsCDC7-CEN HsDBF4-2μ*	MDY95 *YCplac111h-HsCDC7* YEplac181-*HsDBF4*	This study
CY4501	*cdc7Δ ScCDC7-URA3 HsCDC7-2 μ HsDBF4-2μ*	MDY95 YEplac181h-*HsCDC7* YEplac181-*HsDBF4*	This study
CY4245	*cdc7Δ HsCDC7-CEN HsDBF4-2μ*	*MATa his3Δ1 leu2Δ0 ura3Δ0 cdc7::KanMX* YCplac111h-*HsCDC7* YEplac181-*HsDBF4*	This study
CY4246	*cdc7Δ ScCDC7-URA3 HsCDC7-2 μ HsDBF4-2μ*	*MATa his3Δ1 leu2Δ0 ura3Δ0 cdc7::KanMX* YEplac181h-*HsCDC7* YEplac181-*HsDBF4*	This study
CY4481	*cdc7Δ dbf4Δ ScCDC7-LEU2 ScDBF4-URA3*	*MATa his3Δ1 leu2Δ0 ura3Δ0 cdc7::KanMX dbf4::NatMX* YCplac111-*ScCDC7* YCplac33-*ScDBF4*	This study
CY4240	*cdc7Δ dbf4Δ HsCDC7-CEN*	*MATα his3Δ1 leu2Δ0 ura3Δ0 cdc7::KanMX dbf4::NatMX* YCplac111h-*HsCDC7* YEplac181-*HsDBF4*	This study

	HsDBF4-2μ		
CY4242	*cdc7Δ dbf4Δ HsCDC7-2 μ HsDBF4-2μ*	*MATα his3Δ1 leu2Δ0 ura3Δ0 cdc7::KanMX dbf4::NatMX* YEplac181h-*HsCDC7* YEplac181-*HsDBF4*	This study
MDY214	*cdc7Δ dbf4Δ ScCDC7-ScDBF4-URA3*	*MATα his3Δ1 leu2Δ0 ura3Δ0 cdc7::KanMX dbf4::NatMX* YCplac33-*ScCdc7-ScDBF4*	This study
CY4507	*cdc7Δ dbf4Δ ScCDC7-ScDBF4-URA3 HsDBF4 2μ HsCDC7 CEN*	*MATα his3Δ1 leu2Δ0 ura3Δ0 cdc7::KanMX dbf4::NatMX* YCplac33-*ScCdc7-ScDBF4* YEplac181-*HsDBF4* YCplac111h-*HsCDC7*	This study
CY4508	*cdc7Δ dbf4Δ ScCDC7-ScDBF4-URA3 HsDBF4 2 μ HsCDC7-2μ*	*MATα his3Δ1 leu2Δ0 ura3Δ0 cdc7::KanMX dbf4::NatMX* YCplac33-*ScCdc7-ScDBF4* YEplac181-*HsDBF4* YEplac181h-*HsCDC7*	This study
CY5536	*cdc7Δ dbf4Δ ScCDC7-ScDBF4-URA3 HsDRF1-CEN HsCDC7 CEN*	*MATα his3Δ1 leu2Δ0 ura3Δ0 cdc7::KanMX dbf4::NatMX* YCplac33-*ScCdc7-ScDBF4* YCplac111-*HsDRF1* YCplac111h-*HsCDC7*	This study
CY5537	*cdc7Δ dbf4Δ ScCDC7-ScDBF4-URA3 HsDRF1-CEN HsCDC7 2 μ*	*MATα his3Δ1 leu2Δ0 ura3Δ0 cdc7::KanMX dbf4::NatMX* YCplac33-*ScCdc7-ScDBF4* YCplac111-*HsDRF1* YEplac181h-*HsCDC7*	This study
CY5538	*cdc7Δ dbf4Δ ScCDC7-ScDBF4-URA3 HsDRF1-2 μ HsCDC7 CEN*	*MATα his3Δ1 leu2Δ0 ura3Δ0 cdc7::KanMX dbf4::NatMX* YCplac33-*ScCdc7-ScDBF4* YEplac181-*HsDRF1* YCplac111h-*HsCDC7*	This study
CY5539	*cdc7Δ dbf4Δ ScCDC7-ScDBF4-URA3 HsDRF1-2 μ HsCDC7 2 μ*	*MATα his3Δ1 leu2Δ0 ura3Δ0 cdc7::KanMX dbf4::NatMX* YCplac33-*ScCdc7-ScDBF4* YEplac181-*HsDRF1* YEplac181h-*HsCDC7*	This study
CY5628	*cdc7Δ/cdc7Δ dbf4Δ/dbf4Δ HsCDC7-CEN HsDBF4-2 μ*	*MATa/α his3Δ1/his3Δ1 leu2Δ0/leu2Δ0 ura3Δ0/ura3Δ0 cdc7::KanMX/cdc7::KanMX dbf4::NatM/dbf4::NatMX* YCplac111h-*HsCDC7* YCplac181-*HsDBF4*	This study
CY5627	*cdc7Δ/cdc7Δ dbf4Δ/dbf4Δ ScCDC7-ScDBF4-URA3*	*MATa/α his3Δ1/his3Δ1 leu2Δ0/leu2Δ0 ura3Δ0/ura3Δ0 cdc7::KanMX/cdc7::KanMX dbf4::NatM/dbf4::NatMX* YCplac33-*ScCdc7-ScDBF4*	This study
MDY270	*cdc7Δ/cdc7Δ dbf4Δ/dbf4Δ ScCDC7-ScDBF4-URA3 HsCDC7-CEN HsDBF4-2 μ*	*MATa/α his3Δ1/his3Δ1 leu2Δ0/leu2Δ0 ura3Δ0/ura3Δ0 cdc7::KanMX/cdc7::KanMX dbf4::NatM/dbf4::NatMX YCplac33-ScCdc7-ScDBF4* YCplac111h-*HsCDC7* YCplac181-*HsDBF4*	This study
YSC1178-7499555	TAP-Cdc7	*MATa his3Δ1 leu2Δ0 ura3Δ0 CDC7-TAP HIS3*	(GHAEMMAGHAMI *et al.* 2003)
MDY201	TAP-Cdc7 myc^{9}-HsDbf4	*MATa his3Δ1 leu2Δ0 ura3Δ0 CDC7-TAP HIS3* YCp88-*myc*^{9}-*HsDBF4*	This study
YSC1178-7499753	TAP-Dbf4	*MATa his3Δ1 leu2Δ0 ura3Δ0 DBF4-TAP HIS3*	(GHAEMMAGHAMI *et al.* 2003)
MDY200	TAP-Dbf4 myc^{9}-HsCdc7	*MATa his3Δ1 leu2Δ0 ura3Δ0 DBF4-TAP HIS3* YCp88-*myc*^{9}-*HsCDC7*	This study

MDY265	*cdc7Δ ScCDC7-URA3 CDC7-S1*	*MATa his3Δ1 leu2Δ0 ura3Δ0 cdc7::KanMX* YCplac33-*ScCDC7* *YEplac181h CDC7-S1*	This study
MDY266	*cdc7Δ CDC7-S1*	*MATa his3Δ1 leu2Δ0 ura3Δ0 cdc7::KanMX* YEplac181-*CDC7-S1*	This study
MDY267	*cdc7Δ ScCDC7-URA3 CDC7-S2*	*MATa his3Δ1 leu2Δ0 ura3Δ0 cdc7::KanMX* YCplac33-*ScCDC7* YEplac181-*CDC7-S2*	This study
MDY268	*cdc7Δ ScCDC7-URA3 CDC7-S3*	*MATa his3Δ1 leu2Δ0 ura3Δ0 cdc7::KanMX* YCplac33-*ScCDC7* YEplac181-*CDC7-S3*	This study
MDY269	*dbf4Δ ScDBF4-URA3 CDC7-S1 HsDBF4 2 μ*	*MATα his3Δ1 leu2Δ0 ura3Δ0 dbf4::KanMX* YCplac33-*ScDBF4* YEplac181h-*CDC7-S1* YEplac181-*HsDBF4*	This study
MDY317	*cdc7Δ/cdc7Δ dbf4Δ/dbf4Δ ScCDC7-ScDBF4-URA3 CDC7-S1 ScDBF4-LEU2*	*MATa/α his3Δ1/his3Δ1 leu2Δ0/leu2Δ0 ura3Δ0/ura3Δ0 cdc7::KanMX/cdc7::KanMX dbf4::NatM/dbf4::NatMX* YCplac33-*ScCdc7-ScDBF4* YCplac111h-*CDC7-S1* YCplac33L-*ScDBF4*	This study
MDY318	*cdc7Δ/cdc7Δ dbf4Δ/dbf4Δ CDC7-S1 ScDBF4-LEU2*	*MATa/α his3Δ1/his3Δ1 leu2Δ0/leu2Δ0 ura3Δ0/ura3Δ0 cdc7::KanMX/cdc7::KanMX dbf4::NatM/dbf4::NatMX* YCplac111h-*CDC7-S1* YCplac33L-*ScDBF4*	This study
