# Supplementary material for: A Synthetic Human Kinase Can Control Cell Cycle Progression in Budding Yeast
Source: G3 (Bethesda). 2011 Sep 1;1(4):317–25. doi: 10.1534/g3.111.000430 (PMC3276143; doi:10.1534/g3.111.000430)
Supplement: Supporting Information [file supp_1.4.317_TableS3.pdf]

Table S3   Accession numbers of Cdc7 sequences in Figure 8

Species Name	Accession Number
Drosophila melanogaster	NP_727103
Patiria pectinifera	BAI68101.1
Xenopus laevis	AAD21532.1
Danio rerio	NP_001007410.1
Ailuropoda melanoleuca	XP_002926239.1
Bos taurus	DAA31396.1
Homo sapiens	AAC52080.1
Pongo abelii	XP_002810658.1
Macaca mulatta	XP_002801693.1
Callithrix jacchus	XP_002801693.1
Rattus norvegicus	NP_001101822.1
Cricetulus griseus	AAK29327.1
Mus musculus	AAH80702.1
Anolis carolensis	XP_003220149.1
Meleagris gallopavo	XP_003208745.1
Schizosaccharomyces pombe	NP_596328.1
Trichophyton rubrum	XP_003237969.1
Arthroderma gypseum	XP_003173222.1
Candida albicans	AAW28083.1
Pichia pastoris	CCA37102.1
Candida glabrata	XP_448304.1
Saccharomyces kluyveri	SAKL0C07876g
Lachancea thermotolerans	XP_002554175.1
Kluyveromyces waltii	Kwal_27.9804 s27
Vanderwaltozyma polyspora	XP_001645808.1
Zygosaccharomyces rouxii	XP_002494590.1
Saccharomyces castellii	Scas_718.72 s2003
Saccharomyces cerevisiae	NP_010267.1
Saccharomyces bayanus	Sbay_542.8 c542
Kluyveromyces lactis	XP_452391.1
Ashbya gossypii	NP_985073.1
